# Relationship between metabolic syndrome and follicle-stimulating hormone in postmenopausal women

**DOI:** 10.1097/MD.0000000000029216

**Published:** 2022-05-13

**Authors:** Suk Woo Lee, In Sun Hwang, Gyul Jung, Hee Jin Kang, Yoo Hyun Chung

**Affiliations:** a Department of Obstetrics and Gynecology, Hallym University Sacred Heart Hospital, Hallym University College of Medicine, Anyang, Republic of Korea; b Department of Obstetrics and Gynecology, Daejeon St. Mary's Hospital, College of Medicine, The Catholic University of Korea, Republic of Korea; c Department of Obstetrics and Gynecology, College of Medicine, The Catholic University of Korea, Seoul, Republic of Korea.

**Keywords:** follicle-stimulating hormone, insulin resistance, metabolic syndrome, obesity, postmenopause

## Abstract

Depletion of ovarian reserve during menopausal transition raises follicle-stimulating hormone (FSH) markedly and menopause is related to an increased risk for metabolic syndrome (MetS). This study examined the relationship between FSH and MetS in postmenopausal women.

We evaluated the anthropometric values, lipid profiles, high-sensitivity C-reactive protein (hs-CRP) level, Homeostasis model assessment for insulin resistance (HOMA-IR), and serum adipokines levels in 219 postmenopausal women. Serum FSH and estradiol levels were significantly lower in the MetS group than in the non-MetS group. An inverse correlation was observed between FSH with body fat mass (BFM), and HOMA-IR, and a positive correlation was found between FSH and adiponectin level after adjustment for age, years since menopause, BMI, and serum estradiol.

The odds ratio for MetS was higher significantly in the lowest quartile of FSH level than the highest quartile of FSH level (odd ratio = 1.32, 95% CI = 1.09–1.75). Our study showed an increased FSH level favored insulin sensitivity with a higher adiponectin and lower HOMA-IR as well as a lower incidence of MetS in postmenopausal women.

These findings suggest a new approach to the role of FSH for regulating energy metabolism and for use as a biomarker of MetS risk in postmenopausal women.

This systematic review is based on published researches, so there is no ethical approval required.

## 1 Introduction

Metabolic syndrome (MetS) is defined as a combination of various conditions that occur simultaneously, including metabolic risk factors, including dyslipidemia, hypertension, abdominal obesity, and insulin resistance.^[1]^

From menopausal transition to menopause, diminished ovarian function leads to hormonal changes characterized by decreases in reproductive hormones including estrogen, progesterone, testosterone, and inhibin B. These endocrinologic changes may result in metabolic dysfunctions such as weight gain, hyperlipidemia, hypertension, insulin resistance, and abdominal fat deposition; such changes not only serve as risk factors for MetS, but also increase the morbidity and mortality of cardiovascular disease (CVD), which is the leading cause of mortality in postmenopausal women.^[2–4]^ Some adipokines secreted by adipocytes, including adiponectin and leptin, are known to be correlated with the development of specific metabolic states and MetS.^[5]^ These hormones affect the various metabolic dysfunctions that occur during menopause.

Meanwhile, follicle-stimulating hormone (FSH), a glycoprotein polypeptide hormone, is one of the main gonadotropins in the human body, and is synthesized and secreted by the gonadotrophic cells in the anterior pituitary gland. The reproductive actions of FSH in women include maturation of ovarian follicles, proliferation, and differentiation of granulosa cells, and secretion of estrogen during the menstrual cycle.^[6]^ The decrease in the ovarian reserve during the transition to menopause results in elevated FSH levels due to negative feedback of estrogen, which is consistent with previously known findings that FSH levels are regulated only by gonadal hormones.^[3]^ However, recent studies have reported the expression of FSH receptors on extra-reproductive organs, including blood vessels, bone, and the liver.^[7–9]^

Because the increased metabolic dysfunctions that occur during menopause coincide with changes in the levels of reproductive hormones, it is hypothesized that FSH may also be involved in these metabolic dysfunctions in postmenopausal women.^[10]^ Although several studies have investigated the correlation between FSH level and MetS, lipid metabolism, dyslipidemia, and diabetes mellitus (DM) in postmenopausal women, the precise role of FSH in these metabolic dysfunctions in postmenopausal women is still poorly understood.^[8,9,11,12]^

We conducted this study to clarify the relationship between FSH level and MetS in postmenopausal women. In order to do so, we have analyzed the associations between FSH and metabolic risk factors such as obesity, abdominal obesity, markers of insulin resistance, and adipokines.

## 2 Methods

### 2.1 Participants

From September 2009 to December 2011, 281 postmenopausal women seen at Saint Vincent Hospital were recruited into the study. The ethics committee of Catholic University approved this study (VC11TASI0022), and each participant was requested to submit an informed consent. The participants were excluded based on the following exclusion criteria: history of type 2 diabetes mellitus or treatment with insulin or oral hypoglycemic agents; current usage of hormone replacement therapy; chemotherapy or pelvic radiotherapy to treat cancer; steroids or anti-obesity agents that are known to influence body fat and body composition; hysterectomy and/or bilateral oophorectomy undergone before menopause; Cushing's disease; thyroid disease; and premature menopause, which refers to a cessation of menstrual periods before 40 years of age. All participants had experienced amenorrhea of at least 12 consecutive months and possessed no other medical causes of amenorrhea or FSH level greater than 30 mIU/mL. Among the 281 postmenopausal women who were recruited into the study, 30 women who were currently receiving hormone therapy (HT) and 16 women who had previously undergone hysterectomy were excluded. Furthermore, 11 women were excluded due to history of type 2 diabetes mellitus or treatment with insulin or oral hypoglycemic agents. Two women who had previously undergone bilateral oophorectomy, 2 women previously treated with chemotherapy, and 1 woman with a history of thyroid disease were also excluded from the study. After application of the exclusion criteria, 219 women aged between 45 and 71 years (mean [standard deviation (SD)] = 56 [5.2] years) were registered in this study.

### 2.2 Clinical and anthropometric data

Participants provided data regarding their years passed since menopause, age, routine health behaviors such as smoking and alcohol consumption, and whether or not the participants had been previously diagnosed with diabetes or hypertension through answering our questionnaires. Those who drank alcohol at least once a week were categorized as alcohol consumers, and those who smoked at the time of the study were categorized as having a smoking habit.

On the other hand, bioelectrical impedance analysis by a body composition analyzer (Inbody 720, Biospace Inc, Seoul, Korea) was used to measure body composition and size. Various data including visceral fat area (VFA), waist-to-hip ratio (WHR), body fat mass (BFM), percent body fat (PBF), and skeletal muscle mass (SMM) were collected to a degree of accuracy of 1.0% coefficient of variation. Blood pressure was checked twice after a 10-min seated rest using a mercury sphygmomanometer, and the average of the two values was calculated for statistical analysis. BMI was calculated by dividing weight (in kg) by height (in meters) squared. Furthermore, MetS was diagnosed based on ≥3 of the following 5 risk determinants, delineated on the 3rd Adult Treatment Panel (ATPIII) criteria: abdominal obesity (waist circumference >88 cm); increased serum triglycerides (≥150 mg/dL); decreased high-density lipoprotein (HDL)-cholesterol (<50 mg/dL) or specific treatment for lipid abnormality; increased fasting glucose (≥110 mg/dL); and increased blood pressure (≥130/85 mm Hg) or taking an anti-hypertensive agent.^[13]^ Waist circumference was replaced by an increased WHR (>0.85) to assess abdominal obesity.^[14]^

### 2.3 Biochemical analysis

To collect blood samples, venipuncture was carried out after overnight fasting, and HDL-cholesterol, total cholesterol, high-sensitivity C-reactive protein (hs-CRP), triglycerides, fasting plasma glucose (FPG) levels were assayed using a Hitachi 7600–110 Automatic Analyzer (Hitachi Co, Tokyo, Japan). Low-density cholesterol (LDL)-cholesterol was calculated using Friedewald's formula {total cholesterol (mg/dL)–HDL-cholesterol (mg/dL)–total triglyceride (mg/dL)/5}. The coefficients of variation of total cholesterol, triglycerides, HDL-cholesterol, fasting glucose, and hs-CRP were calculated to be 2.0%, 2.2%, 2.6%, 2.3%, and 6.75% (intra-assay) and 1.6%, 2.6%, 0.9%, 1.6%, and 7.91% (inter-assay), respectively. Meanwhile, chemiluminescent immunometric assay using Immulite 2000 Insulin (Siemens Healthcare, Washington DC, WA) was used to measure serum fasting insulin, and the coefficients of variation for insulin were 3.7% (intra-assay) and 8.1% (inter-assay). Insulin resistance was extrapolated by homeostasis model assessment of insulin resistance (HOMA-IR) index {Insulin (mIU/mL) × Fasting blood glucose (mg/dL) / 405}. Lastly, the FSH and estradiol levels were measured with a chemiluminescent microparticle immunoassay (CMIA) using the ARCHITECT FSH and estradiol assays (Abbott Architect, Inc, Chicago, IL). The coefficients of variation of FSH and estradiol were 2.8% and, 4.2% (intra-assay) and 4.1% and, 6.6% (inter-assay), respectively.

Whole blood samples were centrifuged at 300 rpm for 5 minutes to separate the serum and plasma, and aliquots were stored at −80°C prior to analysis. The samples were used together to determine the serum adiponectin and leptin levels using a Quantikine Human Leptin Immunoassay (R&D Systems, Inc, Minneapolis, MN) and Human Adiponectin ELISA Kit (Life Technologies Corp, Darmstadt, Frankfrut, Germany). The coefficients of variation were 3.2% and 3.8% (intra-assay), and 3.0% and 5.1% (inter-assay), respectively for leptin and adiponectin.

### 2.4 Statistical analysis

Statistical analyses were carried out using SPSS (version 18.0; IBM Corp, Armonk, NY). All data are presented as median values (interquartile range [IQR]) or frequencies (%), and non-parametric data were logarithmically transformed to ensure normal distribution. Additionally, variables such as years since menopause, serum estradiol, blood pressure, serum triglyceride, fasting insulin, WHR, HOMA-IR, hs-CRP, and serum leptin and adiponectin were also transformed logarithmically before statistical analyses to approximate normal distribution by Kolmogorov–Smirnov test. Serum FSH was also logarithmically transformed, as it was not normally distributed. Lastly, *t* test or chi-square test was utilized to compare the anthropometric, clinical, and laboratory characteristics between the MetS and non-MetS groups, and Fisher's exact test was used if the expected count was <5 (Table 1). The distribution of FSH levels according to the stratification of age and years since menopause were evaluated using the Kruskal–Wallis test (Supplementary table 1).

**Table 1:** Clinical characteristics of study participants.

	MetS (n = 82)		Non-MetS (n = 137)		
Variables	Median (IQR)	min;max	Median (IQR)	min;max	*P*
Age (year)	57.0 (8)	(46,70)	55.0 (5)	(45,71)	.123
Years since menopause (year)^∗^	7.0 (7)	(2,20)	4.0 (7)	(2, 27)	.304
FSH (mIU/mL)^∗^	49.8 (21.0)	(30.6, 90.1)	53.9 (22.6)	(31.3, 113.1)	.047
Estradiol (pg/mL)^∗^	10.0 (6)	(10,38)	18.6 (5)	(10, 152)	.027
BMI (kg/m^2^)	25.4 (3.6)	(20.5, 32.8)	21.9 (3.1)	(16.3, 31.8)	<.001
WHR^∗^	0.92 (0.05)	(0.86, 1.04)	0.86 (0.05)	(0.78, 0.99)	<.001
PBF (%)	36.1 (3.3)	(28.0, 49.8)	31.2 (7.6)	(16.3, 41.1)	<.001
VFA (cm^2^)	113.7 (22.8)	(33.5, 158.0)	88.4 (13.5)	(37.5, 131.8)	<.001
BFM (kg)	21.6 (5.1)	(15.0, 35.7)	16.3 (5.1)	(7.1, 26.4)	<.001
SMM (kg)	20.3 (3.1)	(16.1,28.3)	19.7 (2.7)	(14.7,35.3)	.006
Total cholesterol (mg/dL)	204.6 (59.5)	(122.0, 351.0)	204.5 (49.5)	(128.0, 324.0)	.860
Total triglycerides (mg/dL)^∗^	174.5 (116.0)	(42.0, 509.0)	81.0 (61.0)	(23.0, 189.0)	<.001
HDL-cholesterol (mg/dL)	44.5 (10.0)	(28.0, 66.0)	53.0 (16.7)	(29.0, 97.0)	<.001
LDL-cholesterol (mg/dL)	120.4 (56.1)	(65.2, 237.6)	131.4 (43.7)	(68.0, 232.2)	.102
FPG (mg/dL)^∗^	98.0 (17.2)	(75.0, 213.0)	94.0 (9.7)	(68.0, 114.0)	<.001
Fasting insulin (μIU/mL)^∗^	4.7 (6.0)	(0.1, 46.6)	2.0 (3.4)	(0.1, 18.9)	<.001
HOMA-IR^∗^	1.2 (1.38)	(0.0, 24.51)	0.46 (0.52)	(0.02, 4.06)	<.001
hs-CRP (mg/dL)^∗^	0.09 (0.90)	(0.03, 0.91)	0.06 (0.04)	(0.01,1.24)	.178
SBP^∗^ (mm Hg)	140.0 (10.0)	(100.0, 182.0)	120.0 (20.0)	(90.0, 150.0)	<.001
DBP^∗^ (mm Hg)	81.0 (10.0)	(60.0, 100.0)	72.0 (13.5)	(54.0, 95.0)	<.001
Serum adiponectin (ng/mL)^∗^	14.0 (5.5)	(6.3, 31.1)	13.1 (8.6)	(5.7, 59.0)	.003
Serum leptin (ng/mL)^∗^	9.5 (5.9)	(0.8, 21.4)	8.8 (4.5)	(0.6, 20.0)	<.001
Current alcohol consumption (%)^†^	5 (6.2)		13 (9.6)		.272
Current smoking status (%)^†^	1 (1.2)		2 (1.5)		.687
Treatment for hypertension (%)^†^	31 (38.3)		17 (12.5)		<.001
Lipid-lowering therapy (%)^†^	31 (38.3)		9 (6.6)		<.001

Data are presented as the median (interquartile range) for continuous variables. BFM = body fat mass, BMI = body mass index, DBP = diastolic blood pressure, FPG = fasting plasma glucose, FSH = follicle stimulating hormone, HDL = high-density lipoprotein, HOMA-IR = homeostasis model assessment of insulin resistance, hs-CRP = high sensitivity C-reactive protein, IQR = interquartile range, LDL = low-density lipoprotein, PBF = percent body fat, SBP = systolic blood pressure, SMM = skeletal muscle mass, VFA = visceral fat area, WHR = waist-to-hip ratio.

∗ Values were analyzed after logarithmic transformation.

† Data are presented as number with proportion for categorical variables.

The correlations between the FSH level and the lipid profile, anthropometric profile, glucose and insulin levels, blood pressure, and serum adipokine levels were examined through Pearson's correlation test. Meanwhile, the correlation between the FSH level and alcohol consumption, smoking, therapy for hypertension, and lipid-lowering therapy were examined using Spearman's correlation test (Table 2).

**Table 2:** Coefficients of correlation between body composition parameters, lipid profile, glucose metabolism-related parameters, hs-CRP, blood pressure, adipokines, alcohol consumption, smoking status, hypertension, lipid-lowering therapy and serum FSH level (A1), and partial coefficients of correlations after adjustment for age, years since menopause, BMI, and estradiol (A2).

	A1		A2	
	*R*	*P*	*R*	*P*
Age	−0.042	.270		
Years since menopause^∗^	−0.033	.315		
Estradiol	−0.186	.003		
BMI (kg/cm^2^)	−0.208	.001		
WHR^∗^	−0.127	.030	−0.130	.080
VFA (cm^2^)	−0.113	.048	−0.120	.098
PBF (%)	−0.038	.286	−0.043	.320
BFM (kg)	−0.164	.016	−0.178	.047
SMM (kg)	−0.150	.013	−0.113	.116
TC (mg/dL)	0.000	.498	−0.043	.322
TG (mg/dL)^∗^	−0.033	.312	−0.065	.243
HDL-C (mg/dL)	0.057	.202	0.079	.198
LDL-C (mg/dL)	−0.005	.472	−0.044	.264
FPG (mg/dL)	0.004	.483	0.019	.417
Insulin (μIU/mL)^∗^	−0.195	.024	−0.161	.073
HOMA-IR^∗^	−0.213	.013	−0.177	.043
hs-CRP (mg/dL)^∗^	−0.006	.466	−0.042	.324
SBP (mmHg)^∗^	−0.024	.396	0.044	.310
DBP (mmHg)^∗^	0.060	.255	0.073	.205
Adiponectin (ng/mL)^∗^	0.192	.016	0.168	.034
Leptin (ng/mL)^∗^	−0.151	.046	−0.006	.472
alcohol consumption^†^	−0.027	.376	−0.044	.311
smoking status^†^	0.090	.149	0.060	.251
Treatment for hypertension^†^	0.131	.065	0.108	.111
Lipid-lowering therapy^†^	−0.028	.373	0.053	.273

Statistical analyses by Pearson's correlation test and partial coefficient of correlations. FSH level was analyzed after logarithmic transformation.

∗ Values were analyzed after logarithmic transformation.

† Values were analyzed by Spearman's correlation test and partial coefficient of correlations.

The serum FSH level was divided into quartiles: the first quartile representing the lowest level, and the fourth quartile representing the highest level. Additionally, the odds ratios (ORs) were evaluated through a binary logistic regression model (Table 3) to investigate the occurrence of MetS according serum FSH levels quartiles.

**Table 3:** Association of FSH with the presence of metabolic syndrome: logistic regression analyses were performed to determine the odd ratios (ORs) of metabolic syndrome with regard to FSH quartiles.

	Odds ratio (95% confidence interval), *P*					
Parameters	Q1	Q2	Q3	Q3		
FSH (mIU/mL)	≤41.23	41.29–52.38	52.47–63.00	≥63.70		
Model 1	1.90 (1.47–2.45),	1.16 (1.05–1.29),	1.65 (1.44–2.38),	1		
*P*	.001	.006	.001		1.73 (0.75–3.98),	1
Model 2	1.32 (1.09–1.75),	1.08 (0.85–1.25),	1.03 (0.83–1.39),	1		
*P*	.010	.225	.361			

Since the variables were interrelated, multiple regression analysis was conducted to recognize the most significant variables that influence FSH. The utilized variables incorporated into the model were as follows: age, years passed since menopause, current smoking status, current alcohol consumption, BMI, WHR, VFA, BFM, total cholesterol, total triglycerides, HDL-cholesterol, LDL-cholesterol, fasting plasma glucose, HOMA-IR, hs-CRP, serum leptin and adiponectin, hypertension, and treatment for hyperlipidemia. However, due to their multi-collinearity, PBF and fasting insulin were excluded from multiple linear regression analysis (Table 4). In all statistical analyses, a *P-*value of ≤.05 was used to test for statistical significance.

**Table 4:** Multiple regression analysis with serum FSH as a dependent variable.

Variables	β	Standard error	Standardized β	*P*
HOMA-IR	−6.371	2.409	−0.224	.009
Adiponectin	0.367	2.281	0.205	.024

## 3 Results

### 3.1 Characteristics of the study population

The clinical characteristics were presented in Table 1. The MetS and non-MetS groups comprised 82 and 137 postmenopausal women, respectively. The mean age and years since menopause were 56.3 ± 5.8 years and 7.7 ± 4.8 years in the metabolic syndrome group and 54.7 ± 5.3 years and 6.9 ± 6.4 years in the non-metabolism syndrome group, respectively. Age, years for menopause, weight, BMI, WHR, and VFA are significantly higher in MetS group than non-MetS group. The median values of serum FSH were 49.8 (21.0) mIU/mL in the MetS group and 53.9 (22.6) mIU/mL in the non-MetS group. Serum FSH level was significantly lower in the MetS group than the non-MetS group (*P* *=* .047; Table 1).

Supplementary table 1 showed serum FSH levels according to stratification of age and years since menopause. The associations between serum FSH levels and both age and years since menopause were not significant (*P* = .641 and *P* = .199, respectively).

### 3.2 Association of FSH with anthropometric values, metabolic factors and adipokines

In the correlation analysis, the FSH level correlated negatively with estradiol level, BMI, VFA, SMM, WHR, BFM, fasting insulin level, HOMA-IR, and leptin level and positively correlated with adiponectin level. However, there was no significant difference in the correlation of age and years since menopause with FSH level. The significant negative correlation between FSH level with BFM, and HOMA-IR, and the positive correlation between FSH level and adiponectin level were maintained after adjusting for age, years since menopause, BMI, and serum estradiol. However, the FSH level showed no significant correlation with the lipid profile and hs-CRP level (Table 2).

### 3.3 Association between FSH and metabolic syndrome

The characteristics of the postmenopausal women according to their FSH level quartiles are summarized in Table 3. The quartile ranges of the FSH level among the postmenopausal women were ≤41.23, 41.29 to 52.38, 52.47 to 63.00, ≥63.70 mIU/mL. Binary logistic regression analyses revealed that the OR for MetS was meaningfully higher in the lowest quartile (Quartile 1) than the highest quartile (Quartile 4) after adjusting for estradiol level, BMI, WHR, SMM, BFM, HOMA-IR, adiponectin, and lipid lowering therapy (model 2: OR 1.55, 95% CI [1.17–2.05], *P* = .03, model 3: OR 1.48, 95% CI [1.18–1.82], *P* = .018, respectively; Table 3).

### 3.4 Multiple regression analysis for the association between FSH and metabolic risk factors

Independent variables that affect serum FSH level were verified using a multiple regression analysis. Among the independent variables, the HOMA-IR and adiponectin were the factors most associated with serum FSH level (*P* = .009 and *P* = .024, respectively; Table 4).

## 4 Discussion

The changes in reproductive hormones that occur throughout a woman's life may affect various factors of MetS such as obesity, abdominal adiposity, insulin resistance, hyperlipidemia, and hypertension.^[10,15]^ Studies of the association between MetS and reproductive hormones have been mainly performed in women with polycystic ovarian syndrome (PCOS) and menopause. An increased luteinizing hormone (LH) or LH/FSH ratio and an increased testosterone level are associated with in insulin resistance in women with PCOS.^[16]^ Diminished ovarian function from menopausal transition to menopause leads to change in sex steroid hormones and deleterious metabolic parameters. Estrogens regulate various aspects of glucose and lipid metabolism and adipokines secretion, and estrogen deficiency leads to increased obesity and abdominal obesity, hyperlipidemia, impairment of glucose metabolism, and insulin resistance.^[17–19]^ Circulating adipokines including leptin and adiponectin secreted by adipose tissue modulate energy homeostasis such as obesity, insulin resistance, and metabolic syndrome and underlying FSH change according to menopause stage is associated with change in adipokine levels.^[1,2]^

FSH is one of the main gonadotrophic hormones secreted from the anterior pituitary gland and has important reproductive functions including fertilization and sex-steroid hormone production. The FSH level increases gradually during perimenopause and menopause in direct response to a decreased signal by endogenous estrogen.^[20]^ Aging attenuates the pituitary response to gonadotropin hormone; the FSH level is also attenuated with aging in postmenopausal women.^[21]^ However, studies have also been reported that age does not affect FSH levels, showing inconsistent conclusions (1). In our study, there was no correlation between both age and years since menopause and FSH level.

FSH has hormonal actions via the FSH receptor (FSHr) in reproductive organs, mainly the uterus and ovaries. However, recent studies have shown that the FSHr is also expressed in nonreproductive organs, including blood vessels, the liver, and adipose tissue, consistent with the nonreproductive actions of FSH in these organs.^[22,23]^

Because sex steroid hormones are regulated by FSH, and because FSH has nonreproductive actions in non-reproductive organs as mentioned previously, it is hypothesized that FSH may be involved in metabolic dysfunctions, as are sex steroid hormones. Several studies investigating the effects of FSH on metabolic risk factors including obesity, lipid and glucose metabolism, insulin resistance, lipid profiles and MetS are now being performed.

In several clinical studies, a lower FSH level was observed in overweight and obese fertile women.^[24,25]^ In a longitudinal study of the association between reproductive hormone levels and obesity in menopausal women, obesity and hormone levels differed by menopausal status, and the FSH level was lower in obesity and higher waist circumference groups than in normal weight and low waist circumference groups.^[26]^ Randolph et al reported that obesity inhibits the increase in FSH in the period after the final menstrual period in Caucasian and Black women.^[27]^ Cui et al first reported lipid biosynthesis via FSH and the FSHr in chicken adipose tissue. They demonstrated that FSHr mRNA was expressed in abdominal adipose tissue and that FSH significantly increased abdominal fat weight. They proposed various pathways and genes related with the regulation of lipid synthesis and fat metabolism.^[9]^ Liu et al reported that FSH antibody reduces body fat in all compartments, namely viscera, subcutaneous tissue, and bone marrow by increasing Ucp1 (uncoupled protein 1) in mouse model.^[28]^ Although previous clinical studies have shown that obesity has an inhibitory effect on FSH levels, experimental studies have reached an opposite conclusion. This paradox probably arises from feedback inhibitory effects of estrogen produced by aromatization in fat tissue on FSH production. Our study showed a negative association between FSH level and BMI and BFM but not abdominal adiposity. In light of the aforementioned studies, it may be that FSH has stimulatory effects on lipid synthesis and that FSH may be inhibited by excessive body fat via an unknown mechanism.

Several clinical studies have done for the effect of FSH on insulin resistance and DM in postmenopausal women. Wang et al performed a clinical study of the association of the FSH level with prediabetes and diabetes in postmenopausal women. In their linear regression, after full adjustment for demographic variables, metabolic factors, estradiol and LH, a higher FSH level was associated with lower fasting plasma glucose and HbA1c levels, and higher FSH quartiles were associated with significantly decreased odds ratios of prediabetes and diabetes. The authors concluded that a low FSH level was associated with an increased waist circumference and HOMA-IR and is consequently a risk factor for prediabetes and diabetes.^[12]^ Another clinical study also showed that higher FSH levels were associated with lower prevalence and incidence type 2 DM and fasting insulin levels in postmenopausal women.^[29]^ Our study showed a negative association between the factors of insulin resistance and FSH after adjustment for BMI and the estradiol level like clinical studies mentioned above. However, the mechanism of the development of insulin resistance and DM by FSH has not yet been elucidated.

There is few study which evaluating the effect of FSH on lipid profiles. Song et al performed both clinical and animal studies of the influences of high postmenopausal FSH levels on the lipid profile. They reported that a higher serum FSH level was associated with a higher serum total cholesterol and LDL-cholesterol level in clinical study. They also showed high serum FSH and lipid levels and reduced hepatic LDL receptor expression by interaction with the FSHr in hepatocytes.^[8]^ However, another study of postmenopausal Chinese women showed positive association between FSH and HDL-C and inverse association with TG and LDL-C.^[30]^ Our study showed no correlation between FSH and lipid profile. Further studies are needed to clarify the association between FSH and lipid metabolism.

Our study showed that adiponectin is one of the independent variables of FSH level. Adiponectin plays a role in reducing the risk of metabolic syndrome by increasing insulin sensitivity and anti-inflammatory effect in adipose tissue.^[31]^ Previous studies showed that adiponectin level is negatively associated with estradiol level and positively associated with FSH level.^[32,33]^

Few studies have examined the impact of FSH on MetS. In two clinical studies, Stefanska et al examined the association between FSH and MetS. FSH levels were lower in postmenopausal women with MetS than in women without MetS and were more closely related to MetS than to SHBG.^[34]^ The authors also suggested that the FSH level reflects the probability of MetS similar to or better than other biomarkers such as CRP, adiponectin, and leptin.^[11]^ In a population-based study, Wang et al found a negative association between FSH and the 10-year risk of atherosclerotic cardiovascular disease (ASCVD) in postmenopausal women in China. They also suggested that a low FSH level is a risk factor for or biomarker of CVD.^[30]^ In our study, FSH levels were inversely correlated with the presence of MetS, as also concluded by Stefanska et al, and Wang et al.

The strength of our study is that it is the first to demonstrate an association between FSH and MetS and obesity, lipid profile, blood pressure, insulin resistance, and adipokines, which are commonly known metabolic risk factors. However, our study has several limitations. First, because of the cross-sectional design, we failed to determine the cause and effect relationship between FSH and metabolic risk factors MetS. Second, the small sample size limited our interpretation of the association between metabolic risk factors and FSH. Third, endogenous androgen and SHBG were associated with MetS and type 2 DM in women in a longitudinal population-based study.^[15]^ However, we did not measure other reproductive hormones such as LH, androgens, or SHBG. Although these hormones may influence the FSH level and metabolic risk factors, we do not routinely measure them in postmenopausal women. Fourth, the WHR was substituted for waist circumference in order to establish a diagnostic criteria of MetS. WHR and waist circumference were both utilized as indicators of abdominal obesity, extending the previous findings in a case–control study which indicated that WHR is a risk factor for CVD.^[35]^ Fifth, we used bioelectric impedance analysis (BIA) to measure the VFA. However, previous study reported that magnetic resonance imaging (MRI) is more accurate for measuring VFA than BIA, and BIA is BIA has a problem that cannot differentiate adipose tissue between visceral and abdominal compartments.^[36]^ Measurement of VFA using BIA may be less accurate. Sixth, because GnRH, LH, FSH have pulsatile release pattern, the serum FSH level should be measured at 10- to 15-minute interval for 6 to 24 hours. However, we measured the single serum FSH level. This can reduce the accuracy of the FSH measurement.^[37]^

In conclusion, our results suggest that the FSH level has an inverse relationship with HOMA-IR, BFM, and the incidence of MetS and positive relationship with serum adiponectin level. These results suggest that unknown mechanisms of FSH in relation to energy metabolism, including fat and glucose metabolisms, may shed light on the effects of FSH on metabolic risk factors associated with MetS and cardiovascular disease. Further larger clinical and experimental studies aimed to reveal the mechanism underlying the effects of FSH on energy metabolism, including fat and glucose metabolism and identify the potential of FSH as a biomarker of the risk of MetS and CVD, are needed.

## Acknowledgments

None.

## Author contributions

**Data curation:** Gyul Jung, Suk Lee, yoohyun chung.

**Formal analysis:** Gyul Jung, yoohyun chung.

**Formal analysis:** In Hwang.

**Software:** Suk Lee.

**Visualization:** Hee Kang, yoohyun chung.

**Writing – original draft:** Hee Kang, Suk Lee, yoohyun chung.

**Writing – review & editing:** Suk Lee, yoohyun chung.

## Supplementary Material

Supplemental Digital Content
