# Supplementary material for: Relationship between metabolic syndrome and follicle-stimulating hormone in postmenopausal women
Source: Medicine (Baltimore). 2022 May 13;101(18):e29216. doi: 10.1097/MD.0000000000029216 (PMC9276200; doi:10.1097/MD.0000000000029216)
Supplement: Supplemental Digital Content [file medi-101-e29216-s001.docx]

| Group | | n | median (IQR) | min;max | *p* |
| --- | --- | --- | --- | --- | --- |
| Age (years-old) | |  |  |  | 0.641 |
|  | ≤ 50 | 25 | 52.4 (17.7) | (30.7, 113.1) |  |
|  | 51-55 | 91 | 54.6 (22.2) | (31.4, 91.1) |  |
|  | 56-60 | 60 | 51.7 (18.9) | (31.8, 107.0) |  |
|  | > 60 | 43 | 51.2 (26.7) | (30.6, 79.1) |  |
| Years since menopause | |  |  |  | 0.199 |
|  | ≤ 5 | 94 | 52.6 (23.9) | (30.7,113,1) |  |
|  | 6-10 | 63 | 54.8 (19.8) | (31.8, 107.0) |  |
|  | > 10 | 62 | 50.3 (21.2) | (30.6, 91,2) |  |

**Supplemental Table 1** Serum FSH levels according to the stratification of age and years since menopause

Statistical analyses by Kruskal-Wallis test. Significance at *p* < .05.
